# Linezolid and atorvastatin impact on pneumonia caused by *Staphyloccocus aureus* in rabbits with or without mechanical ventilation

**DOI:** 10.1371/journal.pone.0187187

**Published:** 2017-11-17

**Authors:** Laure-Anne Pauchard, Mathieu Blot, Rémi Bruyere, Saber-Davide Barbar, Delphine Croisier, Lionel Piroth, Pierre-Emmanuel Charles

**Affiliations:** 1 Laboratoire “Lipides Nutrition Cancer”, U.M.R. 1231, I.N.S.E.R.M., U.F.R. Sciences de Santé, Université de Bourgogne, Dijon, France; 2 Service des Maladies Infectieuses et Tropicales, Hôpital F. Mitterrand, Dijon, France; 3 Vivexia S.A.R.L., Gemeaux, France; 4 Service de Réanimation Médicale, Hôpital F. Mitterrand, Dijon, France; National Yang-Ming University, TAIWAN

## Abstract

Pneumonia may involve methicillin-resistant *Staphylococcus aureus* (MRSA), with elevated rates of antibiotics failure. The present study aimed to assess the effect of statins given prior to pneumonia development. Spontaneously breathing (SB) or mechanically ventilated (MV) rabbits with pneumonia received atorvastatin alone, linezolid (LNZ) alone, or a combination of both (n = 5 in each group). Spontaneously breathing and MV untreated infected animals (n = 11 in each group), as well as uninfected animals (n = 5 in each group) were used as controls. Microbiological features and inflammation were evaluated. Data are presented as medians (interquartile range). Linezolid alone tended to reduce pulmonary MRSA load in both SB and MV rabbits, but failed to prevent bacteremia (59%) in the latter. Linezolid alone dampened TNF-α lung production in both SB and MV rabbits (e.g., 2226 [789] vs. 11478 [10251] pg/g; p = 0.022). Statins alone did the same in both SB and MV animals (e.g., 2040 [133]; p = 0.016), and dampened systemic inflammation in the latter, possibly through TLR2 down-regulation within the lung. However, the combination of LNZ and statin led to an increased rate of bacteremia in MV animals up to 75%. Statins provide an anti-inflammatory effect in rabbits with MRSA pneumonia, especially in MV ones. However, dampening the systemic inflammatory response with statins could impede blood defenses against MRSA.

## Introduction

Methicillin-resistant *Staphylococcus aureus* (MRSA) in both community and ventilator-associated pneumonia (VAP) is frequent in some countries. The highest clinical success rates did not exceed 57% despite appropriate antibiotics [1].

Experimental evidence suggests that mechanical ventilation (MV) could cause specific lung damage (i.e., ventilator-induced lung injury [VILI]) [2]. Moreover, MV could *per se* modify the efficacy of lung immunity and promote an overwhelming inflammatory state if pneumonia develops [3–6]. Actually, a link has been shown between the magnitude of pulmonary and systemic inflammatory responses and outcomes in VAP patients [7].

Statins are lipid-lowering agents that possess immunomodulatory properties that could be in part mediated by the down-regulation of the toll-like receptors (TLRs), involved in pathogens recognition by immune cells [8, 9]. Among TLRs, TLR-2 is likely to sense the immune response to *S*. *aureus* [10]. Statins could be protective in pneumonia according to some concordant clinical data [11, 12]. In addition, experimental studies have demonstrated a lung-protective effect through VILI attenuation [13, 14]. Outcomes in VAP patients were improved by prior treatment with statins [15]. This remains however a controversial issue [16].

We hypothesized that statins could influence the course of MRSA pneumonia in a rabbit model [17]. We used spontaneously breathing (SB) and ventilated animals treated with linezolid (LNZ), atorvastatin or the combination of both, to assess the effect of statins in both settings (i.e. MRSA pneumonia with or without MV).

## Materials and methods

### Animal and study design

Male New Zealand White rabbits (3.0 [0.2] Kg) were bred in the University of Burgundy animal facility (Dijon, France). Animal use and handling were approved by the local veterinary committee (i.e., Comité d’Ethique de l’Expérimentation Animale Grand Campus Dijon [C2EA– 105]), and were performed according to the European laws for animal experimentation in accordance with current recommendations mentioned in the *Guide for the Care and Use of Laboratory Animals*, National Institutes of Health No. 92–23, revised 1985. Animals were randomized into the SB or MV group within each experiment as follows: control uninfected (SB or MV, n = 5 in each), infected (SB+MRSA [n = 11] or MV+MRSA [n = 11]), statin pretreatment infected (SB+MRSA+Statin or MV+MRSA+Statin, n = 5 in each), LNZ-treated infected group (SB+MRSA+LNZ or MV+MRSA+LNZ, n = 5 in each) and the statin pretreatment LNZ-treated infected group (SB+MRSA+Statin+LNZ or MV+MRSA+Statin+LNZ, n = 5 in each).

### Mechanical ventilation

Under general anesthesia provided by ketamine 3.3 mg/Kg (Panpharma, France) and xylazine 1 mg/Kg (Rompun®, Bayer, Germany), a cuff tube of 2.5 mm (Mallinckrodt**™**, Covidien®, U.S.A.) was inserted through the mouth into the trachea under view control. The animal was connected to a volume-controlled respirator (Servo ventilator 900C, Siemens®, Germany) (tidal volume of 12 mL/Kg with zero end-expiratory pressure [ZEEP], a respiratory rate of 25 bpm and an 0.5 inspired fraction of O_2_). Rabbits were kept anesthetized and paralyzed throughout the experiment with midazolam (0.06 mg/Kg/h) (Hypnovel®, Roche, Switzerland) and cisatracurium besilate (0.3 mg/Kg/h) (Nimbex®, GlaxoSmithKline, U.K). Isotonic serum was infused.

### Methicillin-resistant *Staphylococcus aureus* pneumonia induction

The USA300 PVL- strain of *S*. *aureus* was used. At 48 hours before experimentation began, several colonies were harvested, cultured on MRSA2 agar plates (Biomérieux) and then incubated for 24 hours at 37°C. One colony was inoculated into 10 mL of BHI and was incubated for 6 hours before being cultured for 18 hours. A mean titer 9.5 Log_10_ colony-forming units per milliliter (CFU/mL) was used [17]. In the SB groups, inoculum (0.5 mL) was gently flushed through a catheter briefly introduced into the trachea. The animals were then placed upright for 15s and allowed to go back to their cage before being sacrificed 24 hours later. In the MV groups, animals were subjected to MV for 24 hours before being instilled intrabronchially with MRSA. The animals were then kept under MV for 24 hours before sacrifice. Controls received 0.5 mL of saline.

### Statin pre-treatment

Rabbits were pre-treated with statin (20 mg/Kg, as previously described) (Atorvastatin, Pfizer AG, Zürich) by intra-gastric administration, 72, 48 and 24 hours before infection with MRSA [13, 14].

### Linezolid treatment and concentrations

Linezolid (Zyvoxid®, Pfizer, United States) was delivered (125 mg of LNZ in 10 mL of 5% α-cyclodextrin solution) over 1 day through two subcutaneous injections (50 mg/kg), 4 and 16 hours after bacterial challenge, as previously described [18]. Blood samples were obtained 1, 2, 4, 6 and 12 hours after the first drug injection and stored at -80°C. Linezolid concentrations were then determined by high-performance liquid chromatography (limit of detection = 0.03 mg/L).

### Material harvesting

Blood samples were collected (24 hours before bacterial challenge, and then 0, 12 and 24 hours after) and centrifuged at 1,000 g for 15 minutes at 4°C; the supernatant was divided into aliquots and stored at -80°C for cytokine concentration measurements.

All the animals were sacrificed by an intravenous overdose of thiopental (100 mg/kg) and then exsanguinated. Autopsies were carried out so that the lungs and spleen were aseptically taken. They were harvested for ribonucleic acid (RNA) extraction and microscopic examination. The remaining tissue was homogenized to determine cytokine concentrations and for bacterial culture.

### Histological analysis

A tissue sample of 1 cm^3^ that focused on a macroscopic lesion was fixed in formalin and embedded in paraffin. Five-micrometer sections were obtained and colored with hematoxylin and eosin. After viewing five fields per sector in a blinded manner, each section was assigned a numerical histologic score ranging from 0 to 3 according to the degree of PMN infiltration, hemorrhage, and edema in the interstitial and alveolar spaces [19].

### Microbiological evaluation of pneumonia

Each pulmonary lobe was isolated from the whole lung, homogenized and used for serial ten-fold dilution cultures. The mean pulmonary bacterial concentration was calculated (i.e. mean concentration = ∑ [lobar concentration x lobar weight]/ lobar weights). Spleens were processed similarly. An *S*. *aureus*-positive spleen culture was considered a marker of bacteremia.

### Whole blood assay

Fresh heparinized blood from spontaneously breathing (SB) untreated rabbits (n = 10), rabbits subjected to 24-hour MV as described above (n = 5), or either SB or MV rabbits given atorvastatin (20 mg/kg once a day) 72 hours prior to venipuncture was obtained and diluted 1:2 with RPMI 1640 medium (Gibco^TM^ Life Technologies, Saint Aubin, France). Blood was plated at 120 μL/well in a 96-well plate and incubated for 15 minutes at 37°C. Atorvastatin pills were dissolved in RPMI 1640 medium (10 nmol/mL in final volume), added to whole blood (60 μL/well) and incubated for 24 hours at 37°C. Then, heat-killed *S*. *aureus* (HKSA) were added and the blood was incubated for 24 hours at 37°C [17]. Cell viability was checked (propidium iodide dying assay) and culture supernatants were finally removed and kept frozen at -80°C until cytokine concentrations were determined.

### Cytokine measurements

TNF-α and IL-8 concentrations were measured using rabbit specific enzyme-linked immunosorbent assay (ELISA) kits (Uscn Life Science Inc., Wuhan, China). The lower limit was 15.6 picograms/mL for both TNF- α and IL-8.

### Quantitative Real Time (RT)-Polymerase Chain Reaction (PCR)

RNA was extracted from lung samples using the RNA GenElute kit (Sigma-Aldrich, St Louis, MO, USA). Quantitative PCR was performed using IQ Sybergreen Supermix (Bio-Rad, Hercules, CA, USA). Melting curves were performed to ensure the presence of a single amplicon. The following primers were used: rTlr2, forward 5’-TGT CTG TCA CCG AAC CGA ATC CAC-3’ and reverse 5’-TCA GGT TTT TCA GCG TCA GCA GGG-3’ [20]; and rGapdh, forward 5’-ATG TTT GTG ATG GGC GTG AAC C-3’ and reverse 5’-CCC AGC ATC GAA GGT AGA GGA-3’.

The results are expressed as the fold induction using the ΔCt method since the SB animals were considered as the baseline condition.

### Statistical analysis

Data were expressed as means (standard deviations) or medians (interquartile ranges) if not normally distributed.

Group sizes were determined pragmatically according to previous experiments [6].

Continuous variables between the different treatment arms (i.e., atorvastatin alone, LNZ alone or the combination of both, in either SB or MV animals) were compared using 2-way ANOVA, the non-parametric Mann-Whitney U test or the Kruskall-Wallis test, as appropriate, and *post hoc* corrections for multiple comparisons were applied. The Fisher exact test was used for the comparison of categorical variables.

All tests were two-tailed. A *p* value lower than 0.05 was considered significant.

Prism software was used (GraphPad, San Diego California, USA).

## Results

### Statin treatment prior to pneumonia did not improve linezolid efficacy

**Fig 1:**
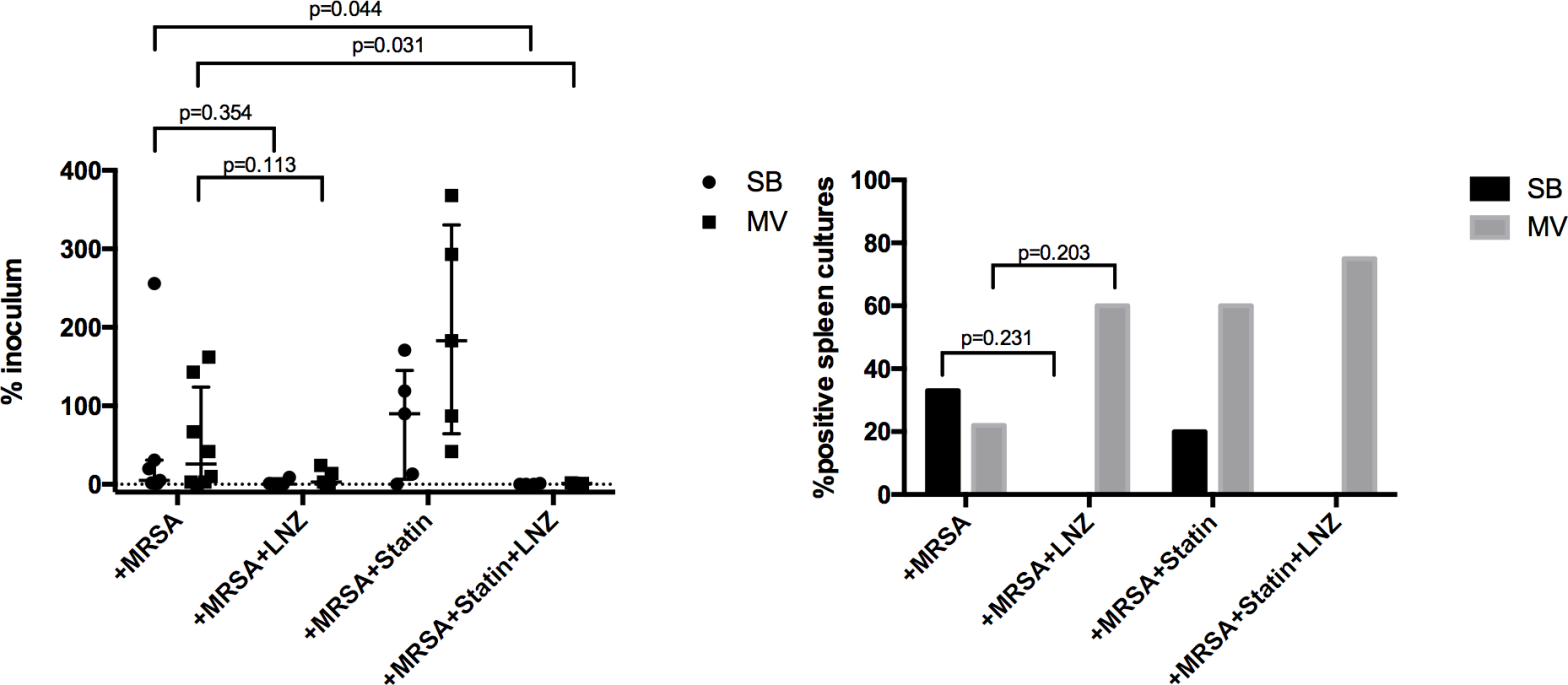
Pulmonary and systemic bacterial burden of *S*. *aureus*. Pulmonary bacterial clearance expressed as the median (IQR) ratio between the amounts of bacteria recovered from the lung culture and the initial *Staphylococcus aureus* inoculum 24 hours after bacterial challenge in either spontaneously breathing or mechanically ventilated animals. Pulmonary-to-systemic bacterial translocation expressed as the spleen positive cultures rate 24 hours after bacteria instillation according to the experimental group in either spontaneously breathing or mechanically ventilated animals with *Staphylococcus aureus* pneumonia. Values are presented as percentages *(right)*. Kruskall-Wallis test with Dunn correction for multiple comparisons or Fisher exact test was used as appropriate for intergroup comparisons. CFU: colony forming unit; MRSA: methicillin-resistant S. aureus; LNZ: linezolid; SB: spontaneously breathing; MV: mechanical ventilation; IQR: interquartile range.

**Fig 2:**
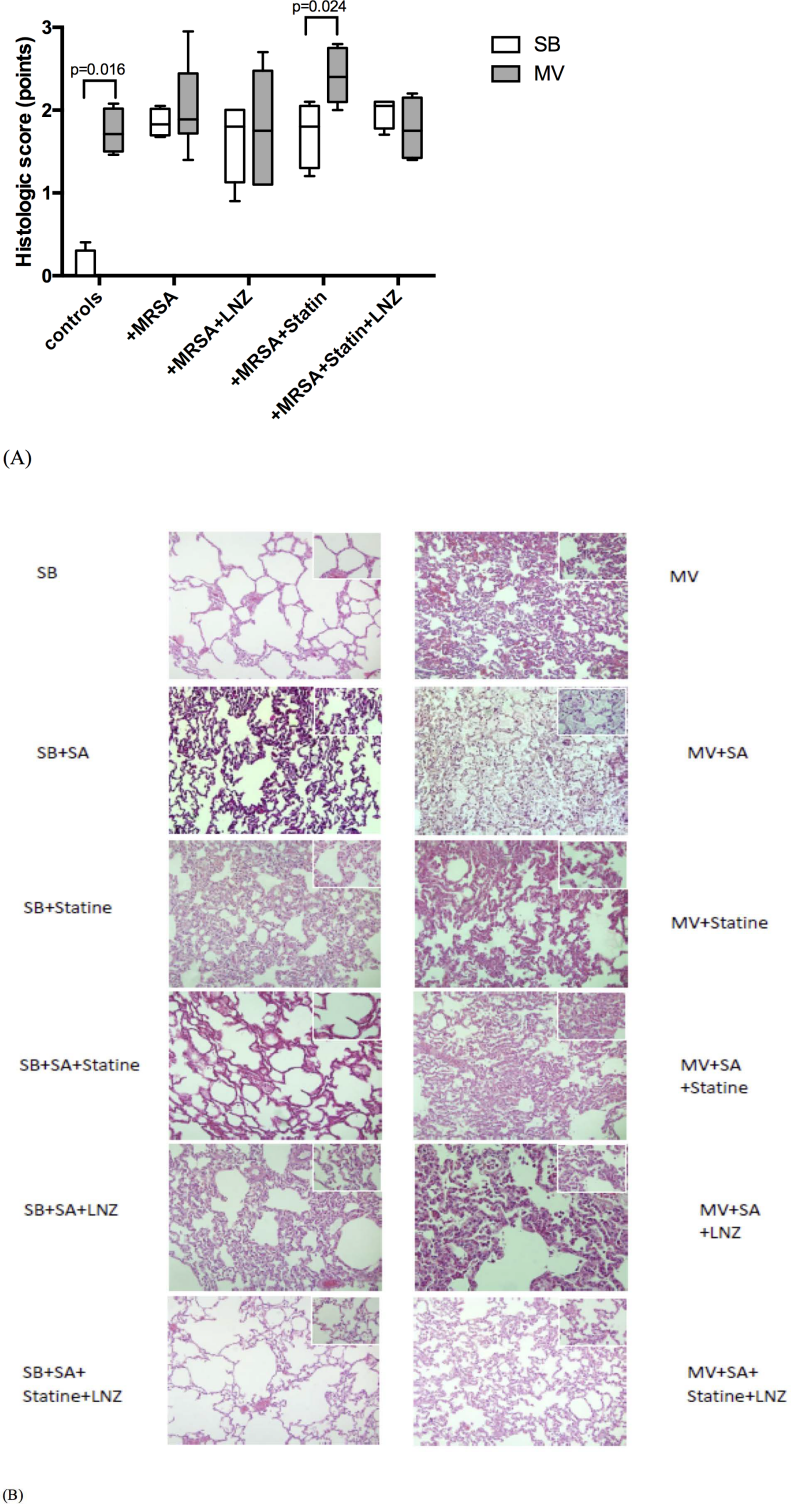
Lung injury main features according to the experimental condition. Histologic score ranging from 0 to 3, based on the degree of polymorphonuklear infiltration, haemorrhage, and oedema in the interstitial and alveolar spaces, following tracheal instillation of saline (controls) or 9 log_10_CFU of *Staphylococcus aureus* in either spontaneously breathing rabbits or animals subjected to mechanical ventilation with various treatment combinations. Mean values (SD) are shown (A) as well as representative light microphotographs of rabbit lungs in various conditions fixed at the same transpulmonary pressure (hematoxylin and eosin x400) (B). Two-tailed Mann-Whitney U test was used for all intergroup comparisons. CFU: colony forming unit; MRSA: methicilline-resistant S. aureus; LNZ: linezolid; SB: spontaneously breathing; MV: mechanical ventilation; SD: standard deviation.

There was a trend toward a reduction of the pulmonary bacterial load in SB and in MV rabbits treated with LNZ, without any improvement in tissue damage (Figs 1 and 2). However, whereas no bacteremia was detected in the SB treated rabbits, LNZ failed apparently to prevent MRSA systemic spillover in MV animals since 59% of them had positive spleen cultures (Fig 1).

Statin therapy alone was associated with a trend towards a greater bacterial burden within the lung, especially in the MV rabbits (183% vs. 26% of the inoculum; p = 0.051), and aggravated lung injury in these animals (Figs 1 and 2).

### Statin treatment prior to pneumonia modified the pulmonary host response

**Fig 3:**
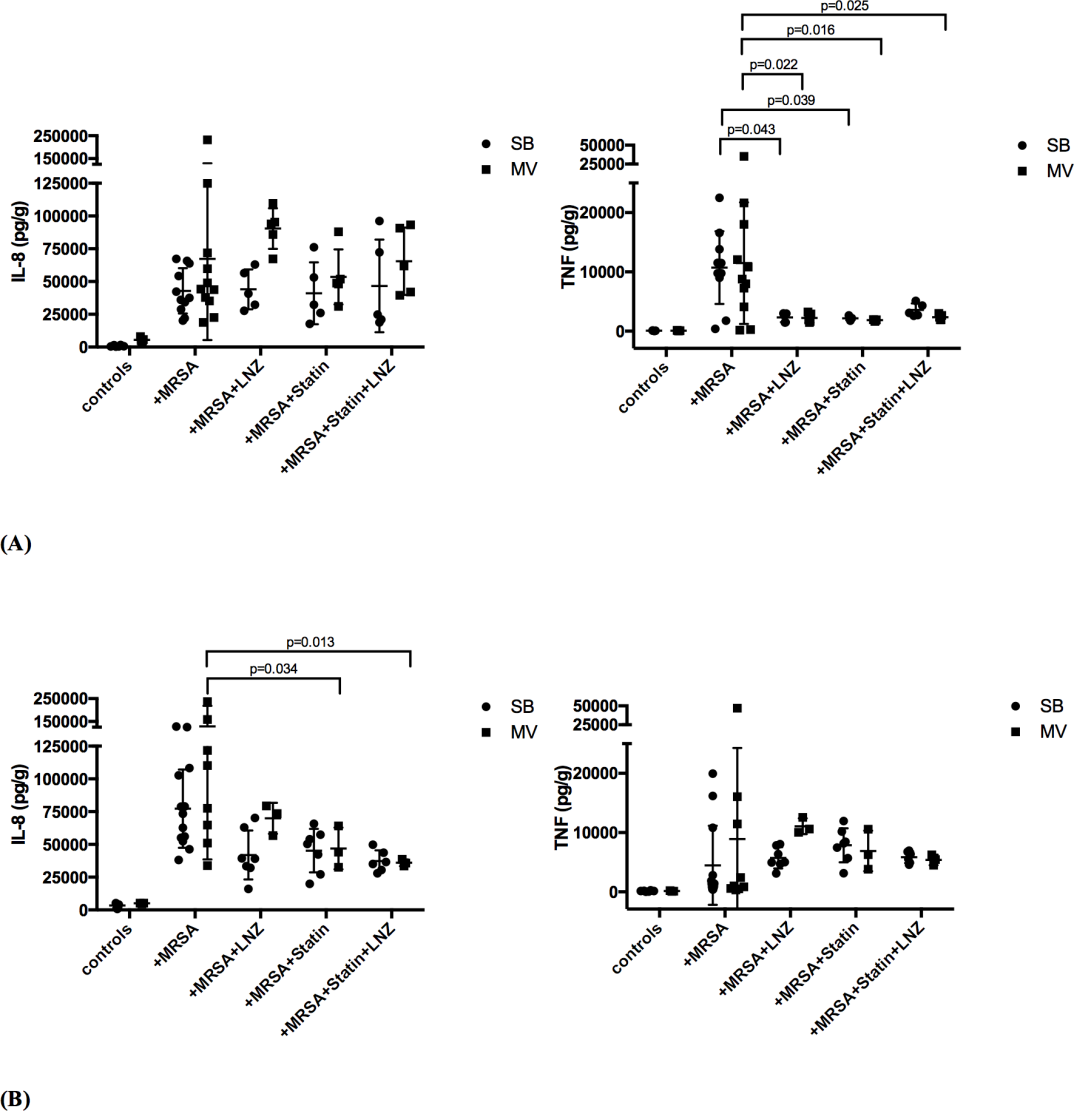
Inflammatory cytokines in the lung and in the spleen of rabbits in various experimental conditions. Inflammatory cytokines (interleukin [IL]-8 and tumor necrosis factor [TNF]-α) concentrations in lung (A) and spleen (B) homogenates 24 hours after tracheal instillation of saline (controls) 10^9^ CFU of *Staphylococcus aureus* in either spontaneously breathing rabbits or animals subjected to mechanical ventilation with various treatment combinations. Values are presented as means (SD). 2-way ANOVA test was used for all intergroup comparisons with Tukey’s adjustment for multiple comparisons. CFU: colony forming unit; MRSA: methicilline-resistant S. aureus; LNZ: linezolid; SB: spontaneously breathing; MV: mechanical ventilation; SD: standard deviation.

Pneumonia development was associated with the release of large amounts of IL-8 and TNF-α within the lung, whether rabbits underwent MV or not (Fig 3).

Linezolid treatment significantly reduced lung inflammation in both the SB and MV groups in terms of TNF-α concentrations (e.g., 2226 [789] vs. 11478 [10251] pg/g in the MV animals; p = 0.022), but not IL-8’s.

Statin treatment alone decreased the release of TNF-α, but not IL-8, in both SB and MV rabbits (e.g., 2040 [133] vs. 11478 [10251] pg/g in the MV animals; p = 0.016).

In animals treated with LNZ, previous statin therapy did not provide an additional anti-inflammatory effect.

### Statin treatment prior to pneumonia modified the systemic inflammatory response

**Fig 4:**
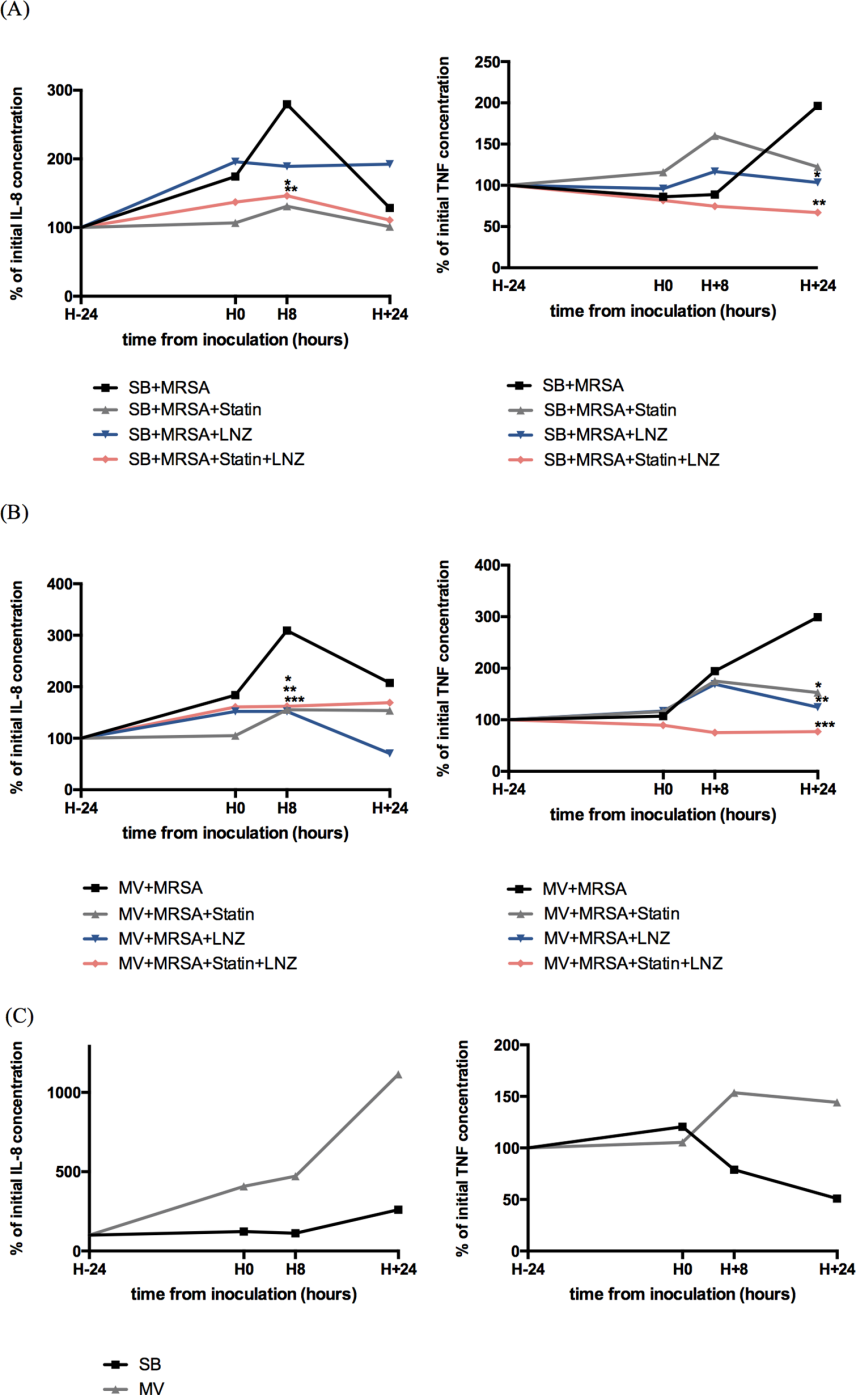
Time course of serum inflammatory cytokine concentrations during experiment. Serum concentrations of interleukin (IL)-8 and tumor necrosis factor (TNF)-α expressed as % of the mean baseline value measured by enzyme-linked immune-sorbent assay at baseline (100%; 24 hours before bacterial airway challenge), just before intra-tracheal instillation of 10^9^ CFU of *S*. *aureus* (H0), and then 8 (H8) and 24 hours (H24) after instillation, in the spontaneously breathing (A) and the mechanically ventilated animals (B). Concentrations measured in non-infected controls are shown in panel C. Variables were compared at each time-point with Mann-Whitney U test and p values were adjusted for multiple comparisons. (A): (*left*) * denotes p = 0.014 between rabbits treated with atorvastatin vs. untreated animals, and ** denotes p = 0.027 between rabbits treated with LNZ+atorvastatin vs. untreated animals; (*right*) * denotes p = 0.049 between rabbits treated with LNZ vs. untreated animals, and ** denotes p = 0.006 between rabbits treated with LNZ+atorvastatin vs. untreated animals. (B): (*left*) * denotes p = 0.004 between rabbits treated with atorvastatin vs. untreated animals, ** denotes p = 0.003 between rabbits treated with LNZ vs. untreated animals, and *** denotes p = 0.014 between rabbits treated with LNZ+atorvastatin vs. untreated animals; (*right*) * denotes p = 0.034 between rabbits treated with atorvastatin vs. untreated animals, ** denotes p = 0.013 between rabbits treated with LNZ vs. untreated animals, and *** denotes p = 0.002 between rabbits treated with LNZ+atorvastatin vs. untreated animals. CFU: colony forming unit; MRSA: methicillin-resistant *S*. *aureus*; LNZ: linezolid; SB: spontaneously breathing; MV: mechanical ventilation. CFU: colony forming unit; MRSA: methicilline-resistant S. aureus; LNZ: linezolid; SB: spontaneously breathing; MV: mechanical ventilation; IQR: interquartile range.

Statin alone reduced IL-8 blood concentrations in MV infected animals (155% [55] vs. 309% [128] of the baseline value in the MV animals; p = 0.032) (Fig 4).

Significantly lower blood levels of IL-8 were achieved in SB rabbits treated with atorvastatin prior to pneumonia and then treated with LNZ 24 hours following the bacterial challenge than in rabbits treated with LNZ alone. The same additive anti-inflammatory effect of statin was observed in MV rabbits according to TNF-α blood levels as early as the 8^th^ hour following the onset of infection (75% [22] vs. 169% [56] of the baseline value; p<0.001).

According to spleen levels of inflammatory cytokines, prior statin treatment alone and the combination of statin and LNZ significantly dampened IL-8 systemic release in MV animals at least, whereas LNZ alone did not (Fig 3).

Of note, MV alone did not influence significantly both IL-8 and TNF- α blood as well as spleen concentrations (Figs 3 and 4).

**Fig 5:**
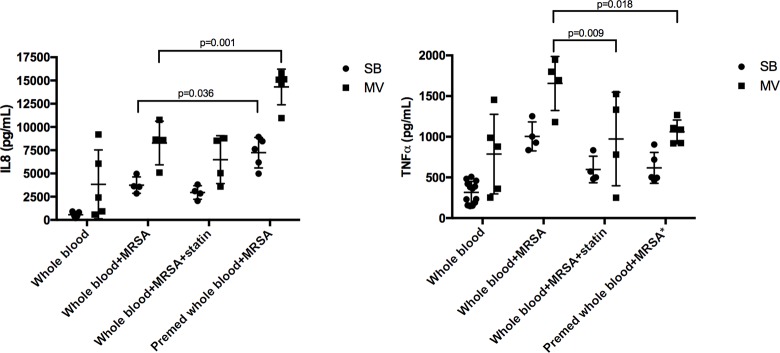
Inflammatory cytokines in whole blood stimulated *ex vivo* by *S*. *aureus* in various conditions. Interleukin (IL)-8 and tumor necrosis factor (TNF)-α mean levels (SD) in the supernatant of whole blood from either spontaneously breathing rabbits or animals subjected to mechanical ventilation. Atorvastatin was added to the medium (“statin”) or given to the rabbit prior to blood sampling (“Premed”). Values are presented as means (SD). 2-way ANOVA test was used for all intergroup comparisons with Tukey’s adjustment for multiple comparisons. One missing [IL-8] and one missing [TNF-α] value in the Whole blood+MRSA and in the Whole blood+MRSA+statin groups. MRSA: methicillin-resistant *S*. *aureus*; SB: spontaneously breathing; MV: mechanical ventilation; SD: standard deviation.

After HKSA *ex vivo* stimulation, levels of both IL-8 and TNF-α were higher in the blood taken from MV rabbits (Fig 5). If MV rabbits were given statin prior to MV, or if the drug was added to the medium prior to *ex vivo* stimulation, TNF-α levels were significantly decreased as low as baseline values.

### Atorvastatin treatment decreased pulmonary and systemic expression of TLR2

**Fig 6:**
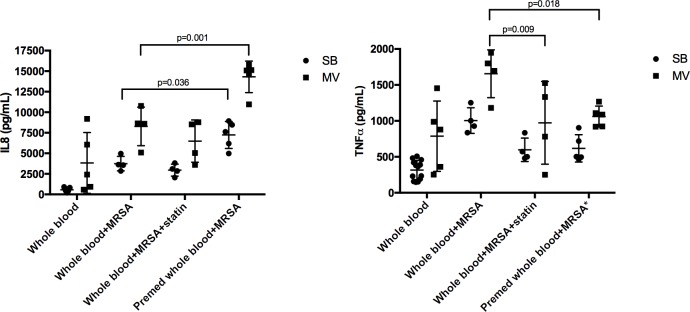
Toll-like receptor 2 gene expression. TLR2 gene expression was measured in both lung (*left*) and spleen (*right*) homogenates in either spontaneously breathing rabbits (SB) or animals subjected to mechanical ventilation (MV) with or without atorvastatin pre-treatment (n = 5 in each group). All values are reported as fold increases compared with SB rabbits. Results are expressed as mean±standard deviation. Kolmogorov-Smirnov test was used for all intergroup comparisons.

Statin treatment decreased *tlr2* expression within the lung of only SB animals (Fig 6), and significantly dampened *tlr2* expression in the spleen of both groups.

## Discussion

We showed herein in a rabbit model of MRSA pneumonia that, although LNZ was efficient in keeping in check bacterial growth within the lung of both SB and MV animals, infection could be considered as more severe in the latter, since pulmonary-to-systemic translocation of *S*. *aureus* was apparently not prevented by LNZ in the ventilated animals. The lung inflammatory response was in part dampened by LNZ in both SB and MV rabbits, but interestingly, an additive effect was achieved by pre-treatment with atorvastatin. Similarly, we showed that LNZ alone failed to completely abrogate the systemic release of inflammatory mediators subsequent to pneumonia, whereas its combination with atorvastatin succeeded in doing so. This anti-inflammatory effect, however, could have hampered the host’s ability to control systemic diffusion of *S*. *aureus*, thereby promoting bacterial spillover into the bloodstream in MV animals.

Cumulative experimental evidence supports the notion that MV itself, especially if adverse settings including large V_T_ are applied, could be harmful in the context of bacterial pneumonia by promoting pulmonary and systemic inflammation [17, 21, 22]. In the present study, we showed that although LNZ therapy was successful in SB rabbits, its efficacy was limited to the lung compartment in MV rabbits. Interestingly, our findings in our model of pneumococci pneumonia treated with moxifloxacin were similar [23]. No significant histological findings were likely to account for such a difference in extra-pulmonary bacterial diffusion, but unseen lung damage is not excluded.

The pulmonary release of TNF-α was reduced by LNZ in both SB and MV rabbits, as was the bacterial burden. It remains unclear as to what extent the anti-inflammatory properties of LNZ *per se* contributed to such a decrease in this key mediator. Actually, experimental studies have shown that LNZ was able to reduce pulmonary inflammation in mice with MRSA pneumonia, in terms of MIP-2 concentrations, thus limiting in turn PMN recruitment and lung injury [24]. In addition, since high levels of IL-8 were still reached despite LNZ therapy, the net effect on lung inflammation is uncertain. Thus, no positive beneficial effect on tissue injury was found.

Treatment with atorvastatin prior to the bacterial infection had anti-inflammatory effects *per se* in the infected lung of both SB and MV animals according to TNF-α concentrations, as reported *in vitro* [9, 25]. It has been shown that human alveolar macrophages incubated with simvastatin released lower amounts of inflammatory mediators in response to a TLR2 agonist, and statin pretreatment decreased lung inflammation following lipopolysaccharide inhalation in volunteers [9, 26].

However, statin therapy alone tended to decrease pulmonary bacterial clearance in MV animals, leading in turn to worse lung injury, thus underlining the need for efficient antimicrobial therapy in combination with the drug. These findings emphasize also the fact that the beneficial effect of statins on VILI is uncertain and should be balanced with their deleterious impact on bacterial clearance [13]. Moreover, the enhanced bactericidal activity of PMNs reported in mice with *S*. *aureus* pneumonia treated with simvastatin is unlikely in our model [27]. Appropriate antibiotics could thus be the prerequisite for any beneficial adjunctive effect of statin therapy in patients with VAP.

The magnitude and persistence of the systemic inflammation are considered key determinants of outcomes in the setting of VAP [7]. Atorvastatin pretreatment could blunt the host inflammatory response within the blood compartment as reported in animal models of sepsis [28]. However, it may be deleterious in our model since bacteremia is an independent predictor of death in MRSA VAP patients [29]. Linezolid might have immuno-modulatory properties likely to dampen MRSA virulence factors, including the expression of pro-inflammatory signals and could thus be beneficial in a mouse model of pneumonia despite persisting bacteremia [30]. Accordingly, the overall effect of the combination of atorvastatin and LNZ could protect the host against a protracted systemic inflammatory response, although delaying *S*. *aureus* clearance from the blood compartment. However, further experimental studies are necessary before any definitive conclusions can be drawn.

Statins exert various immunomodulatory effects but the underlying mechanisms remain unclear [8]. Data from *in vitro* studies suggest that statins could interfere with the TLR pathway, thereby modulating the response to microbial products. However, depending on the experimental model, the net effect of statin treatment could be pro- or anti-inflammatory [25, 31]. For instance, statins could have anti-inflammatory effects through TLR down-regulation [31]. Accordingly, we found that TLR2 gene expression down-regulation by statin within the lung could account for its anti-inflammatory effect. Interestingly, this is in line with previous findings from our group and others showing that *tlr2* up-regulation within the lung of animals subjected to MV probably accounted for overreaction to its agonist including *S*. *aureus* cell wall compounds [6, 32]. In contrast, other mechanisms are probably involved within the blood, suggesting that the spleen was a surrogate for peripheral blood mononuclear cells [33]. Moreover, the effects of statin probably depend on the molecule tested, the cell type and its environment, as well as both dose and duration of treatment prior to stimulation. Herein, we showed that despite one negative effect of atorvastatin treatment prior to pneumonia, namely the systemic release of IL-8 in MV rabbits, *ex vivo* experiments showed that whole blood taken from pretreated MV rabbits overreacted to HKSA stimulation in terms of IL-8 production. These findings, as well as *in vitro* studies, illustrate to what extent impact of statin on the inflammatory response is equivocal.

Although experimental findings support the protective impact of statins in the setting of VAP, strong clinical evidence is still lacking. Actually, the only large multicenter RCT published so far showed that atorvastatin had no effect on the outcome of patients with suspected VAP [34]. However, we have shown that statin treatment prior to VAP was an independent predictor of ICU survival [15]. Similarly, it has been shown by one RCT that overall mortality was reduced in patients with the most severe VAP who received statin prior to infection, as already demonstrated previously in another trial, which included patients with sepsis from various origins [35, 36]. Thus, one cannot exclude the possibility that statins could be beneficial to the host only if given prior to the infection, as was the case in our model. Several explanations could be proposed: (i) more than one administration of the drug is required before critical concentrations in the blood and lung are reached; (ii) the clinical suspicion of VAP is probably later than the actual onset of pneumonia (i.e., airway challenge with bacteria triggering the immune response); (iii) immunomodulation has to occur very early during sepsis in order to prevent any overwhelming pro-inflammatory response.

**Fig 7:**
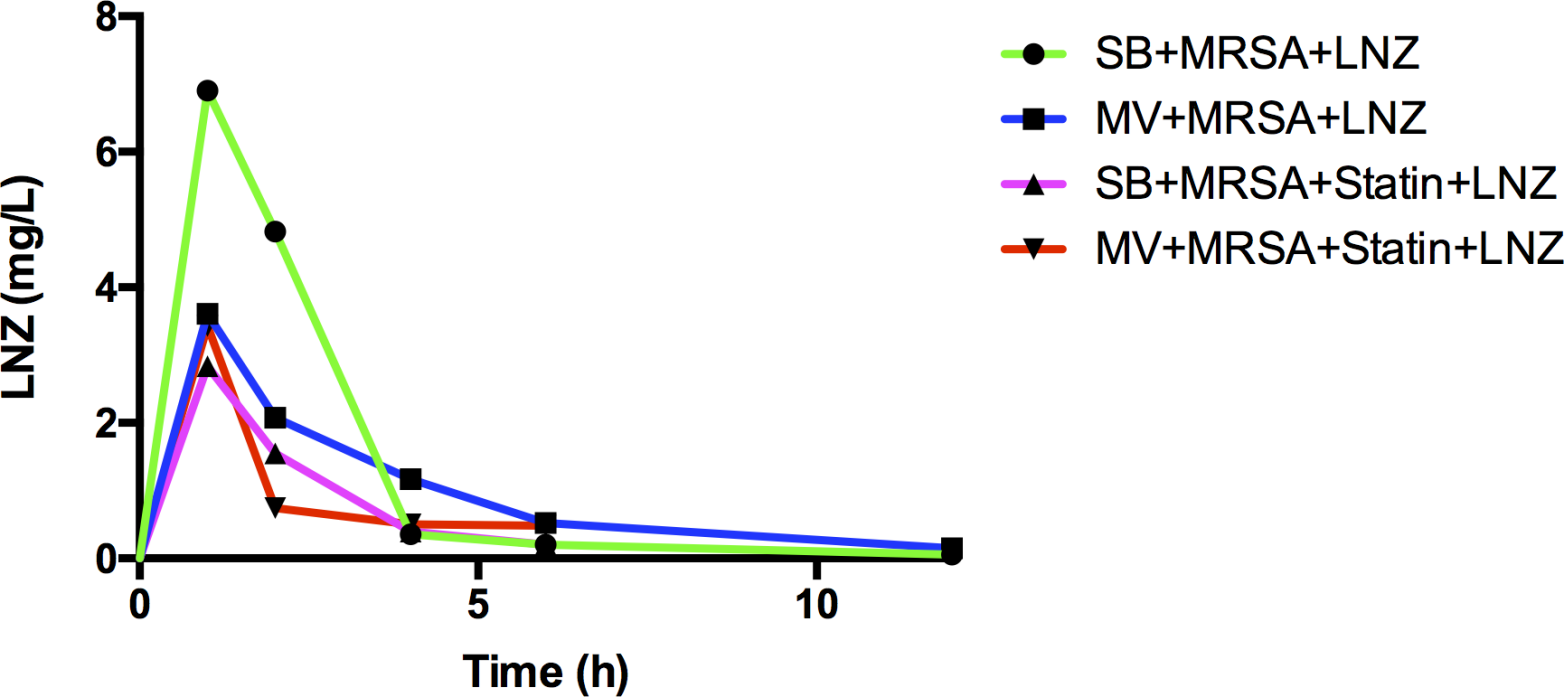
Concentration-time profile for linezolid in rabbits submitted to various experimental conditions. Two-tailed Mann-Whitney U test was used at each time-point for all intergroup comparisons. MRSA: methicillin-resistant *S*. *aureus*; LNZ: linezolid; SB: spontaneously breathing; MV: mechanical ventilation.

Several limitations of our study should be mentioned. First, the MV with “high” V_T_ and without PEEP we used could be considered not clinically relevant. However, it has recently been reported that such settings were still used, especially in the operating room [37]. In addition, it is known that lung injury is heterogeneous in ARDS patients. As a result, poorly aerated areas of the lung usually coexist with overstretched ones, even if “low-V_T_” is applied, as shown in human studies [38]. It is therefore quite possible that such parts of the lung may react as described in the present study if bacterial challenge occurred in patients subjected to “protective” MV. Third, TLR2 assessment was limited to gene expression, and the corresponding protein was not measured. Fourth, our study may be underpowered given the small size of the experimental groups. In addition, we should acknowledge that the LNZ concentrations achieved in the treated animals did not reach the usually reported values in humans, thereby undermining the clinical relevance of our findings (Fig 7). However, we used a MRSA strain, for which the MIC for LNZ was low (i.e., 1 mg/L). In addition, it has recently been shown that the LNZ concentration actually obtained in critically ill patients, especially those with lung injury, were probably not as high as those in healthy volunteers [39]. Finally, our model does not exactly mimic the pathophysiology of VAP since a large number of bacteria were directly inoculated into the airways rather than repeatedly inhaled in small numbers. In addition, there was no mortality attributable to pneumonia in our model, at least over the 48-hour observation period. As a result it remains difficult to evaluate the clinical relevance of our findings.

## Conclusions

Atorvastatin treatment prior to pneumonia provides an anti-inflammatory effect within the lung and the systemic compartment of rabbits with MRSA pneumonia treated by LNZ. This effect may be mediated at least in part by TLR2 down-regulation. Whether such an impact is beneficial for the host remains unclear.

## Supporting information

S1 File Main raw data and statistical analysis reports. (ZIP)
